# Concordance between genome-wide cfDNA screening and diagnostic test results for large copy-number variants: a multi-site study from the Global Expanded NIPT Consortium

**DOI:** 10.3389/fgene.2026.1744417

**Published:** 2026-03-24

**Authors:** Erica Soster, Kristin Dalton, Michael Bonifacio, Katie Battese Ellis, Tristan Hardy, Abdelkader Heddar, Monika Jurkowska, Pascale Kleinfinger, Marizela Kulisic, Kelly Loggenberg, Melody Menezes, Allesio Mori, Giovanni Savarese, Thomas Westover, Sucheta Bhatt

**Affiliations:** 1 Genetics and Women’s Health, Labcorp, Westborough, MA, United States; 2 Medical Affairs, Illumina, Inc., San Diego, CA, United States; 3 Genea, Sydney, NSW, Australia; 4 Monash IVF Group, Melbourne, VIC, Australia; 5 Laboratoire National de Santé, National Center of Genetics, Dudelange, Luxembourg; 6 Genomed SA, Warsaw, Poland; 7 Laboratory CERBA, Saint-Ouen-l'Aumône, France; 8 Next Biosciences, Johannesburg, South Africa; 9 AMES, Centro Polidiagnostico Strumentale, Srl, Naples, Italy; 10 Department of Maternal Fetal Medicine, Capital Health, Trenton, NJ, United States; 11 Department of Obstetrics and Gynecology, Cooper Medical School of Rowan University, Camden, NJ, United States

**Keywords:** cell-free DNA, copy-number variants, deletions, duplications, genome-wide, noninvasive prenatal testing

## Abstract

Fetal copy-number variants (CNVs) have been associated with a broad range of phenotypes and pregnancy outcomes. Noninvasive prenatal screening using genome-wide cell-free (cf) DNA analysis offers an opportunity to detect fetal CNVs early in pregnancy. This retrospective cohort study evaluated concordance between genome-wide cfDNA screening and diagnostic test results for 276 cases with a single isolated cfDNA-identified CNV ≥7 Mb. Cases for this study were submitted by members of the Global Expanded NIPT Consortium. Eight consortium sites in seven countries contributed cases, with 83% of cases submitted from European sites. Seventy-three of 276 cases (26.5%) had no known high-risk indication for cfDNA screening. Mean and median gestational age at the time of cfDNA blood draw was 13 weeks. A deletion was identified for 124 (44.9%) cases and a duplication for 152 (55.1%) cases. Mean CNV size was 33.4 Mb (median 23.1 Mb, range 7–187 Mb). Diagnostic test results were available for 209/276 cases (75.7%). Concordance between cfDNA screening and diagnostic test results was observed for 49/209 cases (23.4%). Mean and median fetal fraction among concordant cases was 8.8% and 8%, respectively. Among 157 discordant cases, a plausible maternal biological explanation was identified for 21 cases (13.4%). Pregnancy outcome information was limited, but available for 116 (42.0%) cases. Parental testing results were available for 38 (13.8%) cases. For six of 15 concordant cases with parental results, fetal CNVs were secondary to a parental translocation or rearrangement. This study contributes to the growing evidence supporting the use of genome-wide cfDNA screening for detection of large fetal CNVs that could affect the current pregnancy and future reproductive risks as well as identify previously unknown maternal conditions.

## 1 Introduction

Up to 2% of pregnancies with normal ultrasound scans and 7% of pregnancies with ultrasound-identified anomalies carry clinically relevant fetal copy-number variants (CNVs) (Acevedo et al., 2025; Evans et al., 2016; Shaffer et al., 2012; Wapner et al., 2012). Given the importance of fetal CNVs to the health of the pregnancy and fetus, the ability to detect these CNVs early in pregnancy may offer new opportunities for changes in management and improved outcomes (Pynaker et al., 2023).

Professional medical societies support offering prenatal screening and diagnostic testing options for fetal chromosomal abnormalities (American College of Obstetricians and Gynecologists' Committee on Practice Bulletins—Obstetrics et al., 2020; Dungan et al., 2023; Hui et al., 2023). Cell-free DNA (cfDNA) screening, also referred to as noninvasive prenatal testing (NIPT), has the highest sensitivity and specificity among all screening options for common trisomies 21, 18, and 13. cfDNA screening is also widely available for fetal sex and sex chromosome aneuploidies (Gil et al., 2017; Norton et al., 2016; Rose et al., 2022), however, common trisomies and sex chromosome aneuploidies constitute only ∼83% of clinically relevant fetal chromosomal abnormalities (Lefkowitz et al., 2016; Wellesley et al., 2012). Genome-wide cfDNA screening methodologies offer the ability to also screen for CNVs and rare autosomal aneuploidies (RAAs) (Mossfield et al., 2022; Rafalko et al., 2021; Scott et al., 2018; Van Opstal et al., 2018; Wang et al., 2022). Recent studies have documented the ability of genome-wide cfDNA screening to provide clinically relevant information about the current pregnancy, maternal conditions, and future reproductive risks (Rafalko et al., 2021; Faieta et al., 2024; Harasim et al., 2022; Kleinfinger et al., 2020; Pedrola Vidal et al., 2024; Pertile et al., 2021; Soster et al., 2023; Van Den Bogaert et al., 2021; van Prooyen Schuurman et al., 2022).

The Global Expanded NIPT Consortium is a collaboration of healthcare providers and laboratory professionals with experience in genome-wide cfDNA screening. This consortium has previously published outcomes of patients receiving high-risk RAA results by genome-wide cfDNA screening (Mossfield et al., 2022; Soster et al., 2024). In this multi-site, global study, the consortium describes 276 cases with a cfDNA screening result positive for a single isolated CNV ≥7 Mb. Concordance between cfDNA and diagnostic test results were assessed. Details on pregnancy outcomes, where available, were also collected. This study did not evaluate cases with microdeletions or microduplications.

## 2 Materials and methods

### 2.1 Study population

Cases for this retrospective cohort study were requested from clinical and laboratory members of the Global Expanded NIPT Consortium. Cases eligible for submission were those that were cfDNA screen-positive for a single isolated CNV ≥7 Mb, with sample collection between July 2019 and June 2023 in the context of routine cfDNA screening in all-risk or high-risk populations under the protocols of the participating site. A CNV size of ≥7 Mb was established for inclusion in this study because most members of the Global Expanded NIPT Consortium used a cfDNA screening test validated to screen for CNVs ≥7 Mb (Pertile et al., 2021; Illumina, Inc., 2024). Demographic details for each patient were collected during each site’s ordering and reporting processes. Patient data was de-identified prior to analysis for this study. This study received an exemption from the Western Institutional Review Board (WIRB)-Copernicus Group (WCG) Institutional Review Board because the research involved analysis of retrospectively collected de-identified data only and therefore did not meet the definition of human subject research as defined by the United States Code of Federal Regulations 45 CFR 46.102.

### 2.2 Genome-wide cfDNA screening

Site-specific laboratory procedures were used to carry out genome-wide cfDNA screening and analysis. All sites used a massively parallel whole-genome next-generation sequencing approach: six sites used the VeriSeq™ NIPT Solution v2 assay (Illumina, Inc., San Diego, CA, United States) (Pertile et al., 2021; Illumina, Inc., 2024); one site used the Illumina TruSeq Nano 16 sample protocol (Illumina, Inc.) (Illumina, Inc., 2017); and one site used the approach described by Lefkowitz et al. (2016). Details of each method are described in the references cited. In brief, the VeriSeq NIPT Solution v2 IVD assay used a PCR-free workflow for library preparation and paired-end sequencing to detect chromosomal anomalies; the TruSeq Nano 16 sample protocol is a single-end sequencing method; and Lefkowitz et al. used shallow whole-genome sequencing with a single read depth algorithm. All three platforms incorporated fetal fraction estimation using either Y chromosome reads, fragment size variation between maternal and fetal fragments, or statistical modeling.

### 2.3 Diagnostic and clinical outcomes collection and concordance assessment

Follow-up information related to diagnostic testing and clinical outcomes was obtained according to the routine procedures of each laboratory or clinic. Information requested from participating consortium sites included details of confirmatory pre- and postnatal diagnostic test results, findings from ultrasound evaluations, pregnancy outcome details, and parental genomic diagnostic test results.

Cases were considered to have had diagnostic testing if at least one of the following were performed: chorionic villus sampling (CVS), amniocentesis, products of conception (POC) testing, placental biopsy, cord blood collection, or postnatal peripheral blood draw. The types of analyses performed on collected samples included microarray, karyotyping, quantitative fluorescence polymerase chain reaction (QF-PCR), fluorescence *in situ* hybridization (FISH), and uniparental disomy (UPD) studies.

Descriptive categories were used to assign cases as:

- Concordant – Cases for which the CNV detected by cfDNA screening was confirmed by diagnostic analysis, regardless of whether additional CNVs were found by the diagnostic analysis. This category includes cases for which the CNV was confirmed only in the placenta, cases for which cfDNA results suggested a partial duplication and diagnostic testing identified a tetrasomy of that region, and cases for which cfDNA results suggested a q arm duplication of a Robertsonian chromosome and diagnostic testing confirmed a whole chromosome trisomy of that chromosome.
- Discordant – Cases for which diagnostic testing did not identify the CNV detected by cfDNA screening.
- Indeterminate concordance – Cases for which concordance could not be determined because diagnostic test results were either not available or were inconclusive.
- Clinical correlation – Cases for which no diagnostic test results were available but other findings, e.g., known parental translocation or maternal CNV, supported inferences or biological explanations related to the cfDNA findings.

### 2.4 Statistical analyses

Study data were described using counts, rates, and measures of central tendency. Statistical comparisons between subsets of the cohort were performed using chi-square or t-tests, as appropriate for categorical or continuous data, with a *P* value of 0.05 used to determine significance. A logistic regression was used to determine the relationship between CNV size and concordance. Analyses were performed in Microsoft Excel.

## 3 Results

### 3.1 Study cohort

Eight consortium sites, including two sites in Australia and one site each in France, Italy, Luxembourg, Poland, South Africa, and the United States, submitted cases for this study. Two hundred seventy-six cases with singleton pregnancies and a single isolated CNV ≥7 Mb in size were included in this study’s cohort.

Mean and median maternal age at the time of cfDNA blood draw was 35 years (range 19–53). Mean and median gestational age was 13 weeks (range 9–37). Patients were largely ascertained in the first trimester and prior to routine anatomical ultrasound with 64.5% of patients having cfDNA screening ordered prior to 14 weeks of gestation, 82.6% prior to 16 weeks, and 92.4% prior to 18 weeks. Two hundred twenty-nine of 276 (83.0%) cases included in this study were submitted by the four European sites.

During case records review, referral indications were streamlined based on the information provided and to have consistency across sites. Seventy-three of 276 cases (26.5%) had no known high-risk indication for cfDNA screening; 78 (28.3%) cases were of advanced maternal age (Table 1). Ultrasound anomalies were reported at the time of cfDNA blood draw for 8/276 cases (2.9%), including two cases with multiple indications.

**TABLE 1:** Testing indications and conception methods. Information about testing indications and conception methods was available for all 276 cases.

Testing indications, n (%)	
Abnormal ultrasound	6 (2.2)
Advanced maternal age	78 (28.3)
Family history	3 (1.1)
Positive maternal serum screen	44 (15.9)
Other indication*	8 (2.9)
Multiple indications	64 (23.2)
None specified^†^	73 (26.5)

Conception methods, n (%)	
Spontaneous	149 (54.0)
Assisted reproductive technology	23 (8.3)
Unknown	104 (37.7)

*Includes six cases with previous miscarriage, one case of “failures of reproduction,” and one case of myoma. ^†^Includes cases with maternal anxiety, patient preference for testing, general screening, and cases with no indication provided.

### 3.2 cfDNA screening results

**FIGURE 1:**
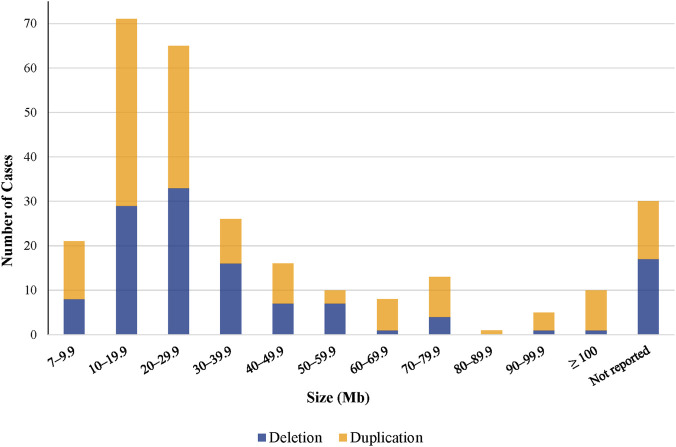
Size distribution of CNVs identified by cfDNA screening (n = 276).

cfDNA screening identified 124/276 cases (44.9%) with a deletion and 152/276 cases (55.1%) with a duplication. CNV size estimations were available for 246/276 cases (89.1%) and ranged from 7 Mb to 187 Mb with a mean of 33.4 Mb and median of 23.1 Mb. Of the 246 CNVs with size information available, 183 (74.4%) were <40 Mb in size while 63 (25.6%) were ≥40 Mb in size (Figure 1). Fetal fraction (FF) estimates were available for 273/276 (98.9%) cases, with a mean FF of 8.9% (median 8%, overall range 2%–25%, interquartile range (IQR) 6%–11%). There was no significant difference in FF between deletion and duplication CNVs.

The chromosomes most frequently found to carry CNVs included chromosomes 5 (n = 32), 10 (n = 28), 7 (n = 27), and 1 (n = 20), comprising 38.8% of the cohort. Recurrent deletions were observed in regions associated with known syndromic deletions such as 1p36, 5p, 6p25, 7q21, 8p23, 11q23, 19p13, and 20q11.2 (Watson et al., 2014). In addition, 17 cases were identified with deletions of the 10q25 region, which has been previously described as a fragile site (Hewett et al., 1998; Huijsdens-van Amsterdam et al., 2018; Zlotorynski et al., 2003).

### 3.3 Diagnostic testing

Diagnostic test results were available for 209/276 cases (75.7%). Among cases with single diagnostic procedures performed, amniocentesis and CVS were the most frequent, comprising 85.2% of procedures. Postnatal testing results were available for 12/209 cases (5.7%). Among cases with single analytic methods applied, karyotype and microarray were the most frequent, together accounting for 54.1% of methods (Table 2).

**TABLE 2:** Diagnostic testing procedures and methods (n = 209).

Diagnostic procedures, n (%)	
Amniocentesis	171 (81.8)
Chorionic villus sampling	7 (3.4)
Cord blood draw (postnatal)	2 (1.0)
Peripheral blood draw (postnatal)	5 (2.4)
Placental biopsy (postnatal)	5 (2.4)
Products of conception	3 (1.4)
Multiple	12 (5.7)
Unknown	4 (1.9)

Analytic methods, n (%)	
Karyotype	41 (19.6)
Microarray	72 (34.5)
Unknown	16 (7.7)
Multiple	80 (38.3)

### 3.4 Concordance

**FIGURE 2:**
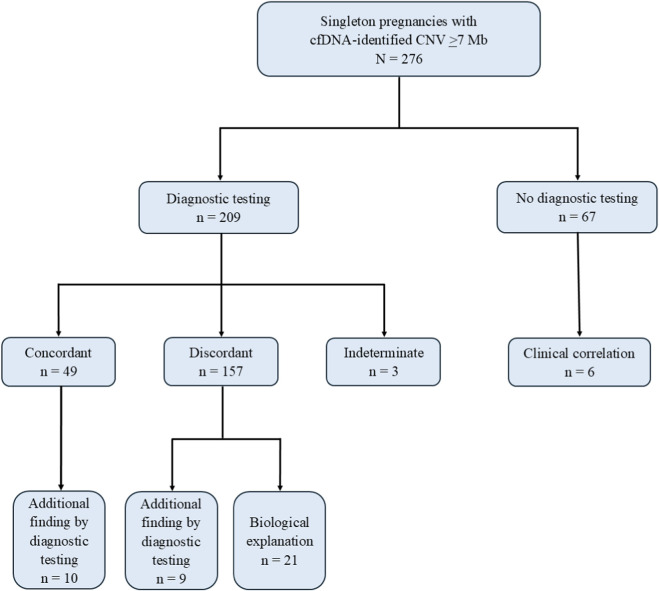
Concordance between cfDNA screening and diagnostic test results for cohort.

Concordance between the cfDNA screening and diagnostic test results was found for 49/209 (23.4%) cases (Figure 2). The mean FF among concordant cases was 8.8% (median 8%, range 4%–21%). Of concordant cases with CNV size estimations available, 34/40 (85%) cases had a CNV <40 Mb in size. For 10 concordant cases, diagnostic testing identified additional CNV findings: for six cases, the additional CNVs were <7 Mb and thereby out of scope for the cfDNA assay due to technical limitations of the cfDNA screening assays used; for four cases, the additional CNVs were ≥7 Mb (in-scope) and potentially detectable by cfDNA (Supplementary Table S1). Of note, 41/49 cases (83.7%) included confirmation by amniocentesis, cord blood, or postnatal peripheral blood (specimens that reflect the genetic status of the fetus rather than the placenta). For the eight remaining cases, the following were observed:

- one case for which the diagnostic test result was concordant, but the diagnostic specimen type was not noted
- two cases for which there was concordant testing on CVS, but no amniotic fluid or neonatal testing was noted
- two cases with testing only on POC but details about the POC specimen types were not provided (e.g., fetal parts, villi, etc.)
- two cases of confirmed confined placental mosaicism (CPM) with the cfDNA result confirmed via CVS, not corroborated by amniotic fluid testing
- one case—a live birth without ultrasound findings—with suspected CPM where the only testing was a postnatal placental biopsy (i.e., no fetal or newborn testing)

For 157/209 (75.1%) cases the cfDNA and diagnostic test results were discordant (Figure 2). The mean FF among discordant cases was 9.1% (median 9%, range 3%–25%). Of discordant cases with CNV size estimations available, 99/141 (70.2%) had CNVs <40 Mb in size. Among the entire subcohort of 157 discordant cases, plausible biological explanations for the cfDNA findings were present for 21 cases (13.4%), including maternal CNV (n = 4), maternal myoma (n = 3), and maternal 10q25 fragile site CNV (n = 14). Additionally, for nine (5.7%) discordant cases diagnostic testing identified CNVs or other chromosomal abnormalities that were different from the CNV identified by cfDNA screening, with eight of nine cases having abnormalities that were <7 Mb and thus out of scope for the cfDNA assay. The remaining case with an in-scope CNV that was not detected by cfDNA screening had an 8.5 Mb deletion on 1p22 identified by karyotype and FISH on amniotic fluid. Details of these 30 discordant cases are shown in Supplementary Table S1.

The difference in concordance rate between deletions and duplications was not statistically significant (*p* = 0.096), however, larger CNVs were less likely to be confirmed than smaller CNVs using a logistic regression model. When comparing cases above and below the median size (23.1 Mb), cases smaller than the median size were significantly more likely to be confirmed than those larger than the median size (*p* = 0.0013).

For the remaining 3/209 (1.4%) cases with diagnostic test results available, concordance could not be determined from the available case data. These cases were categorized as indeterminate concordance. The details of these cases include:

- For one case, cfDNA screening suggested a dup(17) (p11.2p13.3). The diagnostic test result was relayed as ‘abnormal trisomy 17’ with no additional details about the result, specimen type, or procedure type.
- For another case, cfDNA screening suggested a dup(9) (p13.1p24.3). Karyotype on a specimen collected via placental biopsy failed. Although QF-PCR was performed—and results were normal—this assay would not be informative for the chromosome 9 CNV.
- For the last case, cfDNA screening suggested a dup(21) (q11.2q22.13). It was relayed that diagnostic test result was abnormal, but the specific results, specimen type, and procedure type were not shared.

Sixty-seven of 276 (24.3%) cases in this cohort did not have diagnostic test results available (Figure 2). For 61/67 (91.0%) the available clinical information did not support any conclusions about potential relevance of the cfDNA findings and these cases were classified as indeterminate concordance; three of these 61 cases were associated with the known maternal 10q25 fragile site. The remaining 6/67 cases (9.0%) were categorized as clinical correlation. For these cases, clinical information or information from other tests such as parental karyotype or maternal CNV testing suggested potential concordance with the cfDNA findings (Supplementary Table S2).

### 3.5 Pregnancy outcomes and parental testing

Pregnancy outcome information was available for 116/276 (42.0%) cases. Among these 116 cases, there were 77 live births (66.4%), 30 elective terminations (25.9%), and nine miscarriages, intrauterine fetal demises, or stillbirths (7.8%). Detailed pregnancy outcome information was not available for most cases.

Parental testing was documented in 38/276 cases (13.8%); 15 cases were in the concordant group, 14 in the discordant group, five in the clinical correlation group, and four in the indeterminate group. Of the 15 cases in the concordant group, five cfDNA-identified CNVs were secondary to a parental translocation or rearrangement.

## 4 Discussion

In the present study, 276 cases with a cfDNA-identified single isolated CNV ≥7 Mb were assessed for concordance with diagnostic test results and clinical outcomes. Diagnostic test results revealed a concordance rate of 23.4%. Prior studies have similarly reported rates of concordance for cfDNA-identified large CNVs that range from ∼11% to >70% (Rafalko et al., 2021; Wang et al., 2022; Faieta et al., 2024; Kleinfinger et al., 2020; Pedrola Vidal et al., 2024; Pertile et al., 2021; Soster et al., 2023; Van Den Bogaert et al., 2021; van Prooyen Schuurman et al., 2022; Skojo et al., 2024; Soster et al., 2021; van der Meij et al., 2019; Wang et al., 2024; Wang et al., 2021; Wang et al., 2023). Among the 49 concordant cases in this cohort, CNVs spanned a range of sizes and occurred on 18 different chromosomes. Five of the concordant cases—four trisomy 21 and one trisomy 13—were identified as large segmental gains on the VeriSeq NIPT Solution v2 IVD algorithm and classified as full trisomy. This is a known limitation of the IVD platform (Illumina, Inc., 2024). Several CNVs overlapped with known recurrent deletion/duplication syndromes, which could inform counseling about potential phenotypes. In this cohort, most cases were ascertained early in pregnancy, allowing time and opportunity for coordination of confirmatory diagnostic testing, targeted ultrasound evaluations, and other tailored prenatal evaluations and consultations.

Importantly, the vast majority of the confirmed CNVs (83.7%) in the present cohort had diagnostic testing using specimens that reflect the genetic status of the fetus or newborn, rather than the placenta. The remaining cases were confirmed by CVS, POC testing, or in one case, on an unspecified sample type. CPM for CNVs has been previously reported (Sabbagh et al., 2024). In this cohort, CPM was observed in ∼1% of cases with diagnostic testing. Given the limited number of similar cases described in the literature, the clinical consequences of a CNV that is present only in the placenta remain unclear and represent an area for future study.

Although previous studies have shown that cfDNA screening can effectively detect CNVs, test performance metrics vary based on CNV size, methodology used, and sequencing depth (Rose et al., 2022; Lefkowitz et al., 2016; Rafalko et al., 2021; Wang et al., 2022; Pertile et al., 2021; Wang et al., 2021; Kucharik et al., 2020; Liu et al., 2016; Yin et al., 2015; Zhao et al., 2015). With regard to CNV size, Wang et al. (2021) reported a 50% detection rate for CNVs <5 Mb and a ∼40% concordance rate for CNVs >10 Mb, while Liu et al. (2016) reported an 88.89% detection rate for CNVs >10 Mb. The Dutch NIPT Consortium TRIDENT studies reported an overall positive predictive value (PPV) of 32% for segmental aneuploidies—CNVs with a size resolution of 10–20 Mb—with a PPV of 50% in a high-risk population (Van Opstal et al., 2018; van der Meij et al., 2019), leading the national screening program in the Netherlands to allow the option of CNV testing for women undergoing cfDNA screening (Skojo et al., 2024).

Of CNVs identified by cfDNA in the present cohort, smaller CNVs were generally more likely to be concordant than larger CNVs. In 2021, Rafalko et al. (2021) suggested that fetal intolerance to larger CNVs might contribute to higher rates of discordance for larger CNVs. Other studies have similarly reported decreasing PPVs as CNV size increases (Wang et al., 2022; Wang et al., 2021; Shen et al., 2024). Although it would be interesting to assess the relationship between CNV size, concordance, and diagnostic specimen type in this cohort, the cohort size and subsequent size of the subgroups does not allow for a meaningful analysis of this relationship. This is an area for future study in a larger cohort with more complete diagnostic testing details. Additionally, future studies are needed to elucidate the relationship between CNV size and PPV. Of note, FF ranges and means were similar between the concordant and discordant cases in this study, suggesting fetal fraction is unlikely to be related to concordance in this cohort.

Several biological explanations have been recognized to cause false-positive cfDNA screening results, including maternal fragile sites (Huijsdens-van Amsterdam et al., 2018). In this cohort, deletions of the 10q25 region were found for 14 cases classified as discordant and three cases with indeterminate concordance. Maternal testing results were not available for any of these cases, however, based on the breakpoints and the existing literature, it is suspected that all 17 cases can be explained by this known maternal fragile site (Hewett et al., 1998; Huijsdens-van Amsterdam et al., 2018; Zlotorynski et al., 2003). To date, there is no known phenotype associated with the 10q25 fragile site, yet the low level of maternal mosaicism in blood can mimic a reasonable fetal fraction on cfDNA screening. As a result, at least one country has moved away from reporting cfDNA-identified deletions associated with this fragile site to providers and patients (Huijsdens-van Amsterdam et al., 2018).

cfDNA screening also has the capacity to provide information related to maternal conditions with potential implications for pregnancy or maternal care as well as future reproductive risks (Rafalko et al., 2021; Faieta et al., 2024; Harasim et al., 2022; Kleinfinger et al., 2020; Pedrola Vidal et al., 2024; Pertile et al., 2021; Soster et al., 2023; Van Den Bogaert et al., 2021; van Prooyen Schuurman et al., 2022). If a maternal (or paternally inherited) germline CNV is found, the current fetus, any future fetus, and each previous fetus/child each have up to a 50% chance of inheriting the CNV. Identification of unbalanced fetal CNVs by cfDNA analysis raises the possibility of a balanced translocation in a parent, which also has implications for recurrence risks for the parent and their relatives. Additionally, maternal CNVs identified by cfDNA screening can sometimes be associated with myomas, myelodysplastic syndrome, or malignancy. Diagnosis of such conditions can inform management of both the current pregnancy and the patient’s future health. Any of these findings are important for patient care and counseling and warrant further clinical investigation when detected. In the present cohort, a plausible maternal biological explanation was present for 13.4% of discordant cases and parental chromosome analysis revealed parental translocations or rearrangements for six cases (Supplementary Tables S1, S2). Because parental testing was only available for 13.8% of the cohort, however, the rate of parental translocations may be underestimated.

Twenty CNVs in 19 cases in this cohort were not found by cfDNA screening but instead were found during diagnostic testing. Such findings can have clinical implications that would need to be considered by the laboratory and referring physicians on a case-by-case basis. Of the 20 CNVs identified by diagnostic testing, 15 (75%) were out of scope for the cfDNA screening assay as they were <7 Mb in size and therefore not expected to be detected by cfDNA. Similar observations have been reported by others (Soster et al., 2023; Kucharik et al., 2020; Guseh et al., 2021; Maya et al., 2022). The five in-scope CNVs found by diagnostic testing included an 11.2 Mb deletion of 4p, an 8.5 Mb deletion of 1p, a 47.0 Mb duplication of 3p that was mosaic on CVS, a 26.3 Mb duplication of 7p, and a 78 Mb deletion of 4q13. Although this study did not assess sensitivity, the potential for additional CNVs beyond those identified by cfDNA is an important counseling consideration and may inform decisions about follow-up diagnostic testing. Given that three-quarters of the additional findings in this cohort were smaller than 7 Mb, many of these CNVs may be below the limit of detection for a karyotype suggesting that microarray may be a preferred follow-up assay for these cases.

Based on our experience with this study cohort, the consortia members suggest the following when confronted with a genome-wide cfDNA screening result suggesting the presence of a clinically relevant fetal CNV: 1) Thorough genetic counseling with a healthcare provider knowledgeable in the biology of cfDNA and the benefits and limitations of an expanded approach; 2) Ideally, microarray testing on amniotic fluid or postnatal cord blood depending on the preferences of the parents. Microarray will provide information about additional CNVs beyond the limit of detection for a karyotype. However, karyotype may also be necessary to determine the structure of CNVs. In some settings, karyotype may be more easily accessible or cost-effective; 3) Depending on the results of the fetal testing, consideration of parental karyotyping to evaluate for the presence of a parental germline rearrangement to help clarify risk for recurrence; 4) Depending on the cfDNA result, the particular CNV(s) in question, and the sequencing data, review the health history of the pregnant patient to assess for a biological explanation for the cfDNA result. If diagnostic testing on amniocytes or cord blood is normal, it is reasonable to offer additional testing on the placenta if the clinical team or the parents wish to explore further to find an explanation for the cfDNA result. The consortia members encourage close communication between ordering healthcare providers and cfDNA screening laboratories, with ordering providers reporting any discordant cases to the laboratory and laboratories investigating discordant cases if possible.

An important advantage of the Global Expanded NIPT Consortium is that it draws on the real-world experience of clinics and laboratories across diverse populations and varied healthcare system structures. Several of the participating sites and countries routinely offer expanded cfDNA screening in the late first trimester to all pregnant patients, regardless of age. In the present cohort, only 25.4% of cases had ultrasound anomalies or multiple referral indications for cfDNA screening and most cases were screened in the first trimester or early second trimester, before routine anatomy scans are traditionally performed. As such, the a *priori* risk of this cohort may be more representative of generalized pregnancy populations than the cohorts of some previous studies (Lefkowitz et al., 2016; Rafalko et al., 2021; Van Opstal et al., 2018; Soster et al., 2023; Soster et al., 2021). Limitations of this study include the small size of the cohort, which limited the power of statistical analyses, and limited information related to pregnancy outcomes, changes in clinical management, and maternal and family history. Although the sequencing platform and data analysis were highly similar across contributing laboratories, bias could be introduced due to subtle methodological differences. Additionally, because of the long duration of this study, it is not clear how many cases represent subsequent pregnancies from the same individuals. Future studies with larger patient populations and standardized patient selection, data collection, and reporting procedures could be better poised to evaluate how cfDNA findings impacted maternal and fetal clinical care and outcomes.

## 5 Conclusion

This study adds to the growing evidence supporting the use of genome-wide cfDNA screening for early detection of large fetal CNVs. In a broad, largely unselected population, this study demonstrated that cfDNA screening can reveal information relevant for the current pregnancy as well as for maternal health and future reproductive risks for both parents. Additionally, parental testing can support the interpretation of cfDNA screening results.

## Data Availability

All data used in this study that can be made publicly available are included in the article and supplementary material. Requests for additional information will be considered in the context of patient privacy laws and ethical obligations. Inquiries should be directed to the corresponding author.
